# Characterizing changes in executive functions and performance in daily activities after chemotherapy: A pre-post mixed-methods study protocol

**DOI:** 10.1371/journal.pone.0314551

**Published:** 2024-12-13

**Authors:** Khawla Loubani, Debbie Rand, Abed Agbarya

**Affiliations:** 1 Faculty of Health Sciences, Occupational Therapy Department, Ben-Gurion University of the Negev, Beer-Sheva, Israel; 2 Clalit Health Services, Haifa, Israel; 3 Faculty of Medical and Health Sciences, Department of Occupational Therapy, Stanley Steyer School of Health Professions, Tel Aviv University, Tel Aviv, Israel; 4 Oncology Unit, Bnai Zion Medical Center, Haifa, Israel; 5 Faculty of Medicine Technion, Haifa, Israel; Sichuan University West China Hospital Department of Urology, CHINA

## Abstract

**Background:**

Impairments in higher cognitive abilities (termed executive functions (EF) are common among individuals with cancer following chemotherapy and may impact their performance of daily activities. Our aim is to better understand the changes in EF and the impact on performance in daily activities of individuals with cancer pre- and post-chemotherapy.

**Methods:**

A convergent parallel mixed-method pre-post experimental design. Qualitative and quantitative data will be collected pre- and post-chemotherapy (12 weeks following chemotherapy commencement). Participants will be adult candidates for chemotherapy who are newly diagnosed with non-central nervous system malignancy, stages I–III. The Canadian Occupational Performance Measure will assess the performance of daily activities; secondary measures include EF, cognitive functioning, fatigue, and emotional well-being. Qualitative data will be collected via open-ended questions. Pre- and post-chemotherapy, quantitative and qualitative data will be analyzed separately and merged into an overall interpretation. It is expected that, pre-chemotherapy, no difficulties in performing daily activities will be revealed. Post-chemotherapy EF impairments will be apparent and their impact on the performance of daily activities will be identified.

**Conclusions:**

Integrating quantitative and qualitative measures will contribute to a comprehensive understanding of the individuals’ cognitive needs and may enable the development of effective interventions to minimize deterioration in daily activities after chemotherapy.

## 1. Introduction

Cognitive impairments are common following chemotherapy in individuals diagnosed with cancer; these impairments may impact their performance of daily activities. Cognitive impairments have been reported in 17% to 70% of individuals post-chemotherapy [1,2], with some reports of impairments already present during cancer treatment [3,4]. Individuals with cancer often report impairments in short-term and working memory, attention, processing speed, learning, and language, and particularly regarding executive functions (EF) [1,5–7], even though the cancer is not in the central nervous system. EF are higher cognitive abilities that are crucial for performing complex daily activities such as shopping, driving, preparing meals, paying bills [4,8], and coping with novel situations [9]. These activities require different EF components, such as strategic thinking, planning, sequencing multiple steps or actions for completing goals, multitasking, and integrating cognitive processes [10]. Such abilities are also essential in order to manage an individual’s medical condition after the diagnosis of cancer and its related treatments. EF impairments may negatively impact the performance of daily activities and quality of life [11–13], even if the changes are subtle [14].

Cognitive and EF impairments might be identified in earlier stages by self-report; therefore, the use of subjective assessments is recommended. The perceived cognitive impairments are reported more frequently than they are identified by objective cognitive assessments [8], possibly preceding objective brain changes as demonstrated by neuroimaging [15]. Forty percent of individuals with cancer reported cognitive impairments prior to chemotherapy [3], specifically difficulties in multitasking, concentration, word-finding, and short-term memory [16]. Results from some studies indicate that the perceived cognitive impairments are strongly associated with emotional symptoms (e.g., depression and anxiety), coping and adjustment issues [17], cancer-related post-traumatic stress [18], and increased levels of fatigue [19]. Subjective assessments are important indicators of the impact of cancer treatment on daily function and quality of life, can help to identify difficulties in resuming premorbid occupations [14], and provide important data to oncology health care professionals [15].

Cognitive impairments are commonly assessed by neuropsychological assessments that do not capture the functional or subjective aspects. Neuropsychological assessments provide specific, objective information regarding the person’s cognitive abilities [20–22]; however, they do not determine how these impairments affect the individual’s performance of daily activities [4,21]. In order to improve the ecological validity [21], performance-based assessments, which assess the impact of cognition on the performance of daily activities, should be used. Performance-based assessments are generally conducted by occupational therapy practitioners who specialize in assessing cognition in performance, along with its context [21]. It is also important to assess how individuals with cancer perceive these deficits [8] and how they perceive its impact on their performance of daily activities. Therefore, a more comprehensive understanding of the objectively and subjectively perceived cognitive impairments, as well as the impact on daily living, is important. This understanding may enable the development of future interventions that maintain performance of daily activities during and following cancer treatment continuum [23].

Therefore, this study aims to better understand the EF changes and their impact on the performance of the daily activities of individuals with non-central nervous system cancers pre- (immediately after the diagnosis of cancer and before the start of chemotherapy) and post- (12 weeks following commencement) chemotherapy. A mixed methods design will be used by merging quantitative and qualitative data to develop this comprehensive understanding.

The specific aims are as follows:

Compare EF (objective and perceived), the performance of daily activities, emotional symptoms and fatigue pre- and post-chemotherapy and identify which of these variables best explains performance of daily activities pre- and post-chemotherapy (quantitative methods).

Explore the perceived changes in EF and performance of daily activities pre- and post-chemotherapy (qualitative methods).

Integrate the quantitative and the qualitative results by building a study diagram that characterizes the changes in EF and its impact on performance of daily activities, pre- and post-chemotherapy.

## 2. Materials and methods

### 2.1. Study design

**Fig 1:**
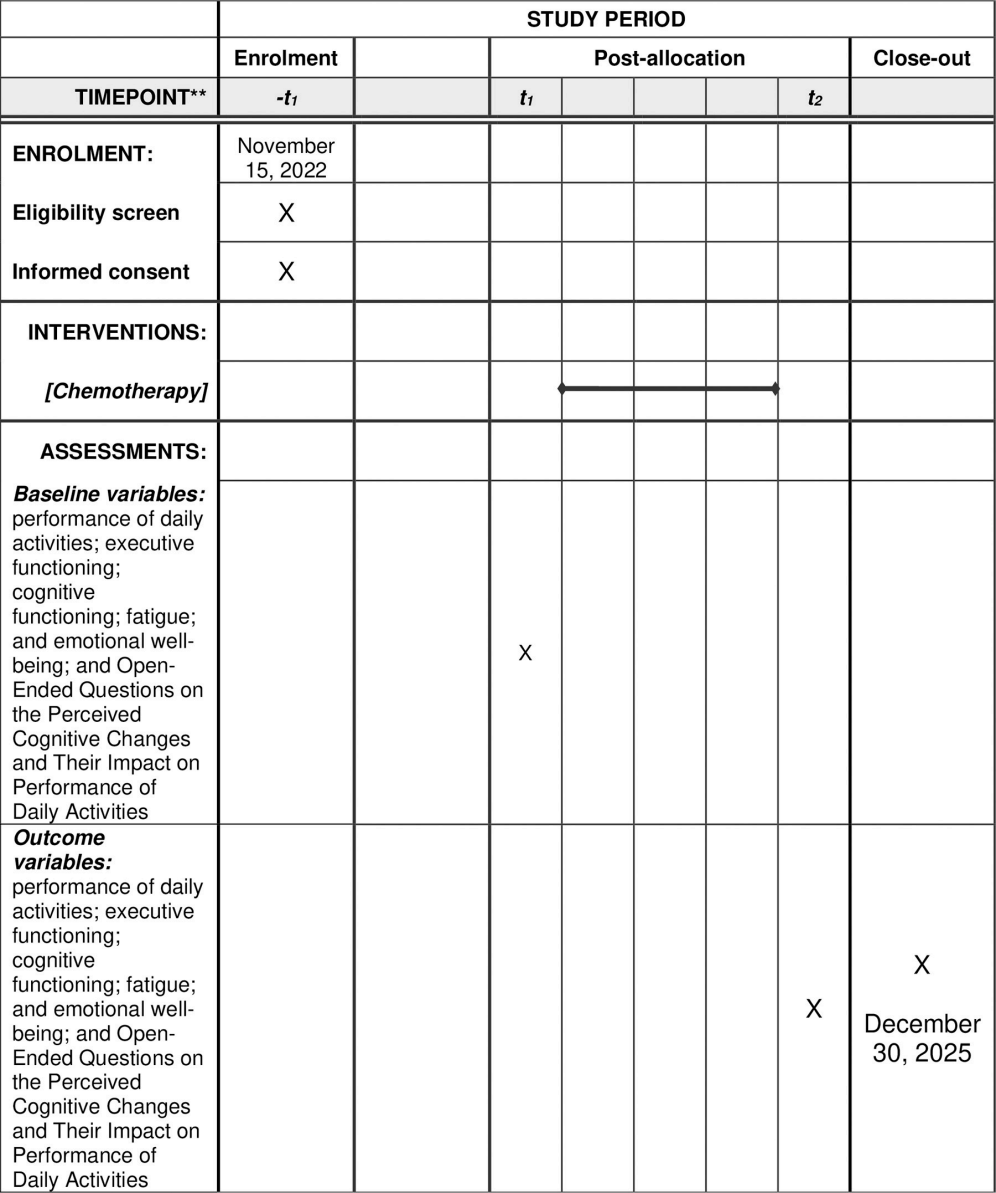
The schedule of enrolment, interventions, and assessments.

**Fig 2:**
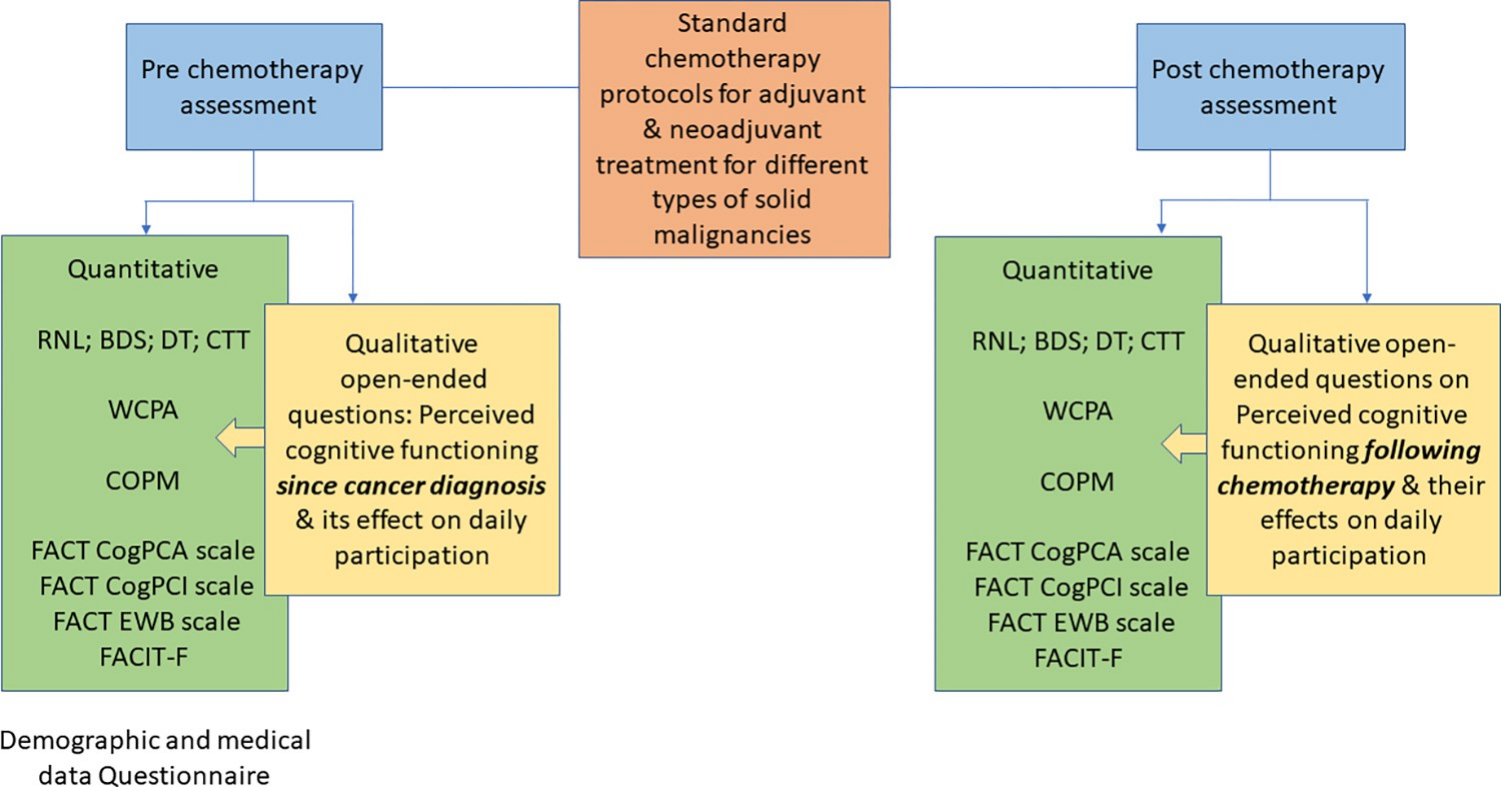
Study design and measures at pre-chemotherapy and post-chemotherapy. Quantitative outcome measures are denoted in green and qualitative outcome measures are denoted in yellow. Abbreviations: BDS = Backward Digit Span; COPM = Canadian Occupational Performance Measure; CogPCA = Cognitive Self Abilities; CTT = Color Trail Test; FACT = Functional Assessment of Cancer Therapy; FACIT = Functional Assessment of Chronic Illness Therapy—Fatigue; EWB = Emotional Well-Being subscale; RNL = Reintegration to Normal Living Index; WCPA = Weekly Calendar Planning Activity.

This manuscript is reported in accordance with the Mixed Methods Article Reporting Standards (MMARS) [24]. The SPIRIT schedule is presented in Fig 1. The pre-post chemotherapy mixed-methods experimental design will enable to understand and compare changes in the participants’ EF and their performance of daily activities between pre- and post-chemotherapy (quantitative). To further understand how changes in EF impact an individual’s performance of daily activities pre- and post-chemotherapy, we utilized a multiple explanatory case study qualitative approach [25] using open-ended questions nested within the pre-post experimental design (Fig 2). Merging the quantitative and qualitative data using a convergent parallel design will enable us to validate and explain the findings from both designs, thus reflecting a comprehensive understanding of the changes that the participants experience [26].

### 2.2. Research questions

The overarching research question is as follows:

How do the changes in EF impact individuals’ performance of daily activities pre- and post-chemotherapy, and how are these changes perceived by them?

#### 2.2.1. Specific quantitative research questions and hypotheses

How do changes in the EF of individuals with cancer impact their performance of daily activities pre- and post-chemotherapy?

1. Daily functioning and performance pre-chemotherapy will be largely explained by emotional symptoms and fatigue.
2. Objective and perceived EF, as well as emotional symptoms and fatigue, will significantly worsen post- compared with pre-chemotherapy.
3. The performance of individuals’ daily activities post-chemotherapy will be largely explained by the objective and perceived EF impairments.

#### 2.2.2. Qualitative research questions

Through the open-ended questions we aimed to better understand how individuals with cancer perceive how changes in EF impact their performance of daily activities pre- and post-chemotherapy. The specific qualitative questions are as follows:

1. Do individuals with cancer perceive changes in their ability to perform complex cognitive tasks?
2. What cognitive difficulties do individuals with cancer experience?
3. What do individuals with cancer think causes these difficulties?
4. How do individuals with cancer perceive that these difficulties can impact their performance of daily activities?
5. What other factors are thought to affect their performance of daily activities?

### 2.3. Participants

Participants will be recruited from the oncology department at a large medical center. The following criteria will determine the participants’ eligibility: adults aged between 18 and 75 years who are candidates for chemotherapy and newly diagnosed with a primary non-central nervous system malignancy including lung, breast, urinary tract, and gastrointestinal cancers (these types will be selected because they are most common), stages I–III pre-commencement of adjuvant (post-surgery), or neoadjuvant (prior to surgery) chemotherapy. They will be required to have proficiency in language, and no previous malignancy, metastatic disease, or neurological conditions that could impair cognitive functions (e.g., dementia, stroke, and brain injury), are not diagnosed with depression, and with at least 12 years of education (which could affect the neuropsychological evaluation). Additionally, hematological cancers will be excluded. All eligible participants will provide written informed consent before enrollment.

The sample size calculation is based on two previous studies [27,28] that used the same primary outcome measure to assess the changes in performance of daily functioning (the Canadian Occupational Performance Measure—COPM). For the current study, a minimum required sample size of 38 participants was determined (calculated by G*Power 3.1 [29], for the Wilcoxon signed-rank test to find differences in matched pairs) with a medium effect size of 0.50, power = 0.80, and α = 0.05, considering an attrition rate of 25%).

### 2.4. Materials and equipment

This study was approved by the Helsinki Committee of Bnai-Zion Medical Center number (BNZ-0106-22) and has been registered on the ClinicalTrials.gov website (NCT05599698).

#### 2.4.1. Intervention

The chemotherapy protocol includes the standard adjuvant or neoadjuvant treatment for different types of solid malignancies, as defined by the oncologist. Such chemotherapy protocols are widely used in the early stages of different types of cancer [30]. Adjuvant chemotherapy is used after a definitive treatment, either surgery or radiotherapy; it is aimed to eradicate any residual micro-metastatic disease in order to reduce recurrences and improve long-term survival. Neoadjuvant chemotherapy was applied before definitive treatment and was primarily used in locally advanced diseases, where a downstaging procedure is mandatory before definitive treatment [31]. Chemotherapy protocols vary according to the chemotherapeutic agent, dose density, and intensity, but are typically administered weekly, biweekly, or tri-weekly for up to six months [30]. The most commonly used agents are 5 Flourouracil, Anthracyclines, Platinum salts, and Taxanes [30]. Participants will come to the oncology department for chemotherapy treatment.

#### 2.4.2. Outcome measures

The following quantitative and qualitative outcome measures will be used for a comprehensive assessment, considering the individual’s personal context, environment, culture and community concerns, valued activities, routines, roles, and previous lifestyle [32].

Quantitative Outcome Measures Pre- and Post-Chemotherapy

1. The primary outcome measure is the Canadian Occupational Performance Measure (COPM) [33], which is an individualized semi-structured interview that assesses performance and satisfaction with one’s performance of daily activities (i.e., self-care, productive, leisure, and social activities). Pre-chemotherapy participants will identify three to five daily activities that they prioritize as meaningful for them; for one person, the performance might be cooking, for another person, it might be walking the dog, and for a third person, it might be participating in a course online [34]. Each of these activities will then be rated on a 10-point scale for perceived performance (1 = not able to do at all, 10 = able to do extremely well) and similarly for satisfaction with performance. The performance and the satisfaction scores represent the average scores for all activities. Post-chemotherapy, participants will rate the same activities they prioritized in the pre-chemotherapy assessment. The COPM has been widely tested in various target groups and its responsiveness (sensitivity to change) is supported in many studies [35–38]. The internal consistency (Cronbach Alpha) in a sample of women after breast cancer was between 0.67–0.79 for performance and between 0.73–0.77 for satisfaction from performance [39].
2. Secondary outcome measures:
  - The Reintegration to Normal Living Index (RNL) [40] will assess participants’ general participation. The RNL consists of 11 statements of participation in recreational and social activities, movement within the community, and the degree of comfort the individuals have in their role in the family and with other relationships. The responses are given on a 1–10-point Likert scale (1, highly do not agree, to 10 highly agree). The adjusted score is calculated as the (Total Score/110) × 100, and ranges from 10–100 points, with a higher score indicating more participation. The RNL has previously been used with patients undergoing surgery and treatment for rectal cancer [41]. It showed acceptable test–retest reliability in different populations (intraclass correlation coefficients (ICCs) ranging between 0.71 and 0.87) [42,43].
  - Objective EF assessments:
    - The Backward Digit Span (BDS) [44] will assess working memory. Participants will be asked to repeat a series of numbers (read out by the examiner) in the reverse order. The number of correct sequences will be recorded. The mean (across age groups) internal consistency reliability of the BDS scores was reported at 0.82 [45].
    - Dual-task assessment will assess the ability to perform multi-tasking. This will include a motor task performed using the hands—the Box and Blocks Test (BBT) [46] and a cognitive task—repetitively counting backwards by subtracting threes. Participants will perform the tasks as single tasks and the two tasks concurrently (motor and cognitive) in order to assess the interference (cost) of the motor task on the cognitive task (cognitive cost) and the impact of the cognitive task on the motor task (motor cost). The BBT is a standardized, reliable, and valid assessment tool used to assess manual dexterity; its test–retest reliability is high (intraclass correlations coefficients of 0.89 to 0.97) [47,48]. Participants will be asked to transfer wooden blocks from one side, over a wooden partition, to the other side of a box within one minute. The number of blocks transferred in 1 minute will be registered. This test will first be performed by the dominant and then by the non-dominant hand. More blocks indicate greater upper-extremity manual dexterity. For the single cognitive task, participants will be asked to count backwards by subtracting the number 3 from a three-digit number (i.e., 399) and then to keep subtracting 3 from their answer for 1 minute. The number of correct answers will be recorded. The motor/cognitive cost calculation is: (single task—dual task)/single task ×100.
    - The Color Trail Test (CTT) [49] is a paper and pencil test. This visual attention timed neuropsychological test will assess cognitive flexibility. The CTT1 consists of 25 circled numbers from 1 to 25 (even numbers are in a yellow background and odd numbers are in a pink background). The participants will be asked to connect the circles in consecutive order, which assesses visual attention and perceptual tracking. The CTT 2 consists of double the stimuli; two sets of 25 numbers in each color (pink and yellow); this part requires divided attention, cognitive flexibility, and more complex perceptual tracking. The participants will be asked to connect the numbers alternating between the two-color sets (1-pink, 2-yellow, 3-pink, 4-yellow…). The completion time will be recorded for CTT1 and CTT2 (up to 240 seconds). In addition, the score of the Interference Index will be calculated as follows: (CTT2 time raw score—CTT1 time raw score)/CTT1 time raw score. The Interference Index score reflects the difference in the participant’s performance between CTT1 and CTT2. Its retest reliability among healthy adults was 0.909 for the CCT-A and 0.912 for CTT-B [50,51].
  - The Weekly Calendar Planning Activity (WCPA) [32], a performance-based assessment tool, will assess the impact of EF on the participant’s ability to perform a multiple-step activity. A list of 10 appointments is presented to the participant, who is asked to schedule them in a 1-weekly calendar while adhering to five rules. The scores include accuracy, the number of appointments entered, planning time, total time, efficiency, error types, the number of rules followed, and the number of strategies used. Interrater reliability ranges between 0.64 to 0.98 supported in a number of studies across ages, cultures, and clinical populations [52,53]. To our knowledge, this is the first study to use WCPA in cancer.
  - The perceived cognitive and executive functioning abilities:
    - Cognitive Self Abilities Scale CogPCA (FACT-Cognitive Function Version 3) [53] (https://www.facit.org/ (accessed on 1 July 2022)) will assess perceived cognitive abilities, including those related to EF. The CogPCA scale includes seven statements with responses given on a 5-point Likert scale (from 0, never, to 4, very much, several times a day). The score ranges from 0 to 28, with higher scores indicating greater difficulties. The CogPCA reliability was found (Cronbach’s alpha coefficient = 0.93) in patients with non-CNS cancers [54].
    - The Perceived Cognitive Impairment scale—PCI (FACT-Cognitive Function Version 3) [54] (https://www.facit.org/ (accessed on 1 July 2022)) will assess the perceived cognitive abilities, including those related to EF. The PCI scale includes 18 statements with responses given on a 5-point Likert scale (from 0, never; to 4, very much, several times a day). The score ranges from 0 to 72, with higher scores indicating greater difficulties. The CogPCI reliability is high (Cronbach’s alpha coefficient = 0.97) in patients with non-CNS cancers [55].
  - The Emotional Well-Being subscale (EWB) [56] from the FACT-G (General) questionnaire (https://www.facit.org/ (accessed on 1 July 2022)) will assess the participants’ emotional status. The EWB scale includes six statements with responses given on a 5-point Likert scale (from 0, not at all, to 4, very much). The score ranges from 0 to 24, with higher scores indicating greater emotional deficits. The EWB subscale was found to be sensitive to change in patients with cancer. Its test-retest reliability within 3–7 days was 0.82 [55].
  - The Functional Assessment of Chronic Illness Therapy—Fatigue (FACIT-F) (Version 4 https://www.facit.org/ (accessed on 1 July 2022)) [57] will assess the participants’ fatigue. The FACT Fatigue includes 13 statements with responses given on a 5-point Likert scale (from 0, not at all, to 4, very much). The score ranges from 0 to 52, with higher scores indicating more fatigue. Internal consistency (Cronbach’s alpha = 0.84) and test–retest reliability (intraclass correlation coefficient = 0.92) was good in a mixed population [58].
  - Demographic and medical data will be collected using a questionnaire with the following details: age, education, sex, marital status, the number of children, the years of education, work status, dominant hand, socioeconomic level (income level), the type/stage of cancer, disease characteristics (e.g., the size of the tumor, the number of affected lymph nodes), the details of the treatments received (e.g., the types of surgery chemotherapy regimens, the date of chemo commencement, and the number of cycles completed).

#### 2.4.3. Qualitative outcome measures

Qualitative data will be collected through open-ended questions pre- and post-chemotherapy. The questions will be printed, and participants will be asked by the assessor to answer in writing (see the questions in Table 1). At the pre-chemotherapy assessment, participants will be asked to describe whether they recognize any changes or difficulties in their cognitive functioning since the cancer diagnosis, whether they feel or recognize the impact of these changes on their performance of daily activities, and how these changes are reflected. Post-chemotherapy, participants will be asked to address the same questions by comparing their condition to the pre-chemotherapy assessment.

**Table 1:** Flow chart of the qualitative open-ended questions pre- and post-chemotherapy.

Open-Ended Questions on the Perceived Cognitive Changes and Their Impact on Performance of Daily Activities			
Pre-Chemotherapy		Post-Chemotherapy	
The tests that you just experienced assessed your cognitive ability. Please describe how it went. How do you usually manage complex cognitive tasks? What did you prefer or dislike**?**		Like our previous meeting, the tests you passed aimed to assess your cognition. Please describe how it went. In comparison to our previous meeting (before chemotherapy), describe whether you feel any difference in your ability to perform complex cognitive tasks.	
**If the answers indicate cognitive difficulties** **(ask the questions below)**	If the answers indicate **“no”** cognitive difficulties (ask the questions below)	**If the answer indicates cognitive difficulties**	If the answer indicates **“no”** cognitive difficulties
Please specify the difficulties. What are the challenges? Why do you think something is wrong? Please specify.	What other factors do you think affect your daily functioning? Please specify.	What are the changes you experience in your cognition? Please specify.	What other factors do you think affect your daily functioning? Please specify.
What do you think causes these difficulties? Please specify.		What do you think causes these changes? Please specify. If chemotherapy is not mentioned as a cause, ask: “Some people report a change in cognition following chemotherapy. Do you feel any changes? If so, please specify.”	
How do you think these difficulties are reflected in your daily functioning? Please specify.		How do you think these changes are reflected in your daily functioning? Please specify.	
What other factors do you think may affect your daily functioning? Please specify.		What other factors do you think affect your daily functioning? Please specify.	
		If chemotherapy is not mentioned as a cause, ask: “Some people report a change in daily functioning following chemotherapy. Do you feel any changes? If so, please specify.”	
**End**			

### 2.5. Procedure

Participants will be recruited by the medical oncology staff. Each participant will provide written informed consent. Assessments will be conducted pre- and post-chemotherapy (Fig 2). Each assessment session will last up to one hour and will be performed by a qualified OT. Assessments will take place in the oncology department of the Medical Center or at the participants’ home. Recruitment will take place from November 15, 2022, to December 30, 2025 (Fig 1).

The objective EF measures and the cognitive performance-based assessment (WCPA) will be administered, followed by the qualitative part and, thereafter, the FACT questionnaires.

### 2.6. Data analysis plan

Pre- and post-chemotherapy, the quantitative and qualitative data will be analyzed separately and then the results will be combined and merged into an overall interpretation.

#### 2.6.1. Quantitative data analysis

At the pre- and post-chemotherapy assessments, data analysis will be performed using SPSS version 27 [59] (https://www.ibm.com/products/spss-statistics (accessed on 16 March 2023)). Descriptive statistics will be used to describe the study population and the outcome measures. Normal distribution of the variables will be verified using the Shapiro–Wilk test. For variables found to be non-normally distributed, appropriate **non-parametric tests** will be applied. In order to handle error variance in explanatory variables (e.g., demographics), we will consider the transformation of each of these variables into dummy variables where necessary, allowing for more accurate regression modelling. To identify the variables that best explain the daily performance (COPM), multiple linear regression analysis (Enter method) will be used pre- and post-chemotherapy. The enter method will enable us to explore how each independent variable contributes to the model, which is particularly valuable in our study due to the exploratory nature of the research when there are no theoretical reasons to enter the variables stepwise or hierarchically [60]. By treating all variables equally, it will help avoid missing important predictors, which is crucial given the novel context of our study [61]. Given that the sample might not be the same (due to attrition), to confirm that differences in explaining variables are not due to the composition of the sample (but rather to the impairments caused by the chemotherapy), we can also run this analysis, for only participants who have pre- and post-assessments. Three variables that will be significantly correlated with daily performance (pre/post) will be entered into the model. Scatter plots of residuals against the model data will be inspected, as well as outliers and influential data points, along with the variance inflation factor for multicollinearity.

To compare the pre- and post-chemotherapy assessments, for normally distributed variables, paired t-tests will be used; when data are not distributed normally and the scales of measurements are ordinal, non-parametric tests will be used; for the primary outcome (COPM, an ordinal scale), the Wilcoxon Signed Ranks test will be used.

#### 2.6.2. Qualitative data analysis

**Fig 3:**
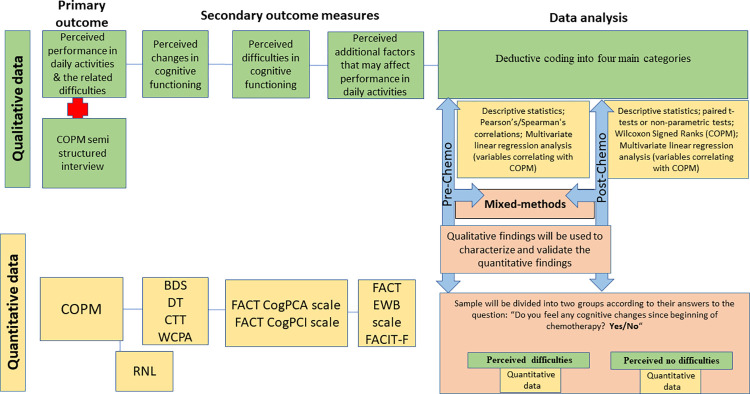
Mixed methods study design for pre- and post-chemotherapy. Abbreviations: BDS = Backward Digit Span; COPM = Canadian Occupational Performance Measure; CogPCA = Cognitive Self Abilities; CTT = Color Trail Test; FACT = Functional Assessment of Cancer Therapy; FACIT = Functional Assessment of Chronic Illness Therapy—Fatigue; EWB = Emotional Well-Being subscale; PCI = Perceived Cognitive Impairment; RNL = Reintegration to Normal Living Index; WCPA = Weekly Calendar Planning Activity.

The qualitative data include the participants’ answers to the open-ended questions at pre- and post-chemotherapy and additional data during the administration of the semi-structured interview of the COPM. Qualitative data will be analyzed using deductive coding, which is particularly relevant for explanatory case studies [62] methods while ensuring the participants’ anonymity by using pseudonyms (e.g., in their quotations). Data will be coded and categorized into four main categories, which have been determined in advance for the coding protocol in accordance with the literature review and quantitative outcome measures (Fig 3): perceived performance in daily activities and related difficulties; perceived changes in cognitive functioning; perceived difficulties in cognitive functioning; and perceived additional factors that may affect performance in daily activities. First, we will revise and/or confirm that the codes do appear in the data by finding examples; we added codes to the codebook if additional codes are identified in the data; finally, we identified the main themes that will arise [63]. Two authors will perform the text analysis deductively and undertake a thorough description and validation by a few participants to ensure its trustworthiness [26].

#### 2.6.3. Mixed methods/merging the data

At pre- and post-chemotherapy, the quantitative results will be reported, followed by qualitative quotes or themes that either support or refute the quantitative results. We will identify qualitative content areas to expand our understanding of the main quantitative results, specifically to those areas that explain how the EF difficulties impact the participants’ performance of daily activities pre-chemotherapy.

Post-chemotherapy, we will divide the sample into two groups (with and without perceived difficulties) according to the participants’ Yes/No answers to the question: “Have you experienced any cognitive changes since you began the chemotherapy?”. The dichotomy of group classification will be performed according to participant’s answers (Yes/No). The changes in EF and performance of daily activities will be compared between groups. In addition, we will build a study diagram to illustrate the results post-chemotherapy.

## 3. Expected results

The expected results will be described separately for each of the assessments pre- and post-chemotherapy (Fig 2).

### 3.1. Pre-chemotherapy

We expect that the participants’ perceived performance (COPM) will approach 10 (the highest level of performance). In addition, we expect that the participants’ secondary objective outcomes will indicate no difficulties regarding general participation (RNL), cognitive functioning (BDS, DT, CTT, WCPA FACT CogPCA, and FACT CogPCI scales), and fatigue (FACIT-F). However, we expect that the emotional outcome measure (FACT EWB) will indicate emotional difficulties, which will significantly explain the variance in daily performance in the regression analysis.

We expect that the participants’ answers to the open-ended questions will validate the quantitative results by reporting no perceived difficulties in their cognitive functioning. In addition, we expect that participants will report no or a minor impact of the cognitive difficulties on their performance of daily activities. To generate the results (mixed methods), we may use tables or figures that display both the quantitative and the qualitative results.

### 3.2. Post-chemotherapy

We expect that the quantitative results would show decreased performance of daily activities, lower COPM scores, and lower secondary quantitative outcomes. We expect that participants will have more difficulties in their cognitive functioning and particularly EFs following chemotherapy and that these difficulties will impact their daily performance and will also be reflected in the qualitative results. The classification of participants into two groups (perceived with or without perceived cognitive difficulties) will enable us to characterize each group by the quantitative findings.

## 4. Discussion

EF impairments may negatively affect the daily functioning of individuals with cancer, even if impairments are subtle [12,14,39]. This mixed methods study design aims to better understand the changes in EF impairments and the impact on the performance of daily activities pre- and post-chemotherapy of individuals with non-central nervous system cancers. Additionally, we aim to understand the effects of other aspects that are known to be associated with perceived cognitive impairments after cancer, such as emotional symptoms and fatigue [17–19].

Our main outcome measure is the COPM, which assesses performance in daily activities relevant to each individual [34]. Using the COPM in this study will facilitate alignment between participants’ needs and priorities and professional clinical judgment [62], as well as suggestions for future cognitive intervention priorities. The results of this study may help oncology personnel to be aware and recognize these impairments at an earlier stage to refer patients to receive occupational therapy services. The results may also be helpful to the interdisciplinary oncology team, which can be involved in the assessment and management of cognitive symptoms. The early detection of individuals with cognitive difficulties pre- and post-chemotherapy will enable providing these individuals with occupational therapy interventions to minimize EF impairments and functional decline [38]. These findings may lead to close communication between occupational therapy and the oncology staff members to enhance performance in the daily activities of individuals with cancer as part of a multidisciplinary approach.

## 5. Expected limitations

Some limitations are expected. The study will be conducted at a single medical and the sample will include only individuals with solid non-CNS cancers. Hematological cancers are outside the scope of our study, which may limit the ability to make conclusions about the effect of chemotherapy in this specific group. The sample might be heterogeneous in terms of age, cancer type, chemotherapy, and premorbid cognitive abilities, however, each participant will be assessed twice (pre-post chemotherapy) and compared to themselves, which will provide insight into the effects of the chemotherapy. Furthermore, it could also be argued that, due to heterogeneity in cancer types and chemotherapy, other side effects or complications associated with chemotherapy may not be covered in this study. Additionally, this study will not examine the effects of a specific chemo agent on EF and daily functioning. Further research should include follow-up assessments at six months and one-year post-chemotherapy.

## 6. Conclusions

This pre-post chemotherapy mixed-method study could potentially lead to the development of effective occupational therapy interventions to minimize deterioration in daily activities after chemotherapy. Future research should include larger, more diverse samples across multiple centers to validate the effectiveness of pre/post-chemotherapy interventions. Findings may lead to close communication between occupational therapy and the oncology staff members as part of a multidisciplinary approach to treat individuals with cancer.

## Supporting information

S1 Checklist SPIRIT 2013 checklist: Recommended items to address in a clinical trial protocol and related documents*. (DOC)

S1 Protocol (DOC)

## Abbreviations

Abbreviation: Full term
EF: Executive Functions
BDS: Backward Digit Span
BBT: Box and Blocks Test
COPM: Canadian Occupational Performance Measure
CogPCA: Cognitive Self Abilities
CTT: Color Trail Test
FACT: Functional Assessment of Cancer Therapy
FACIT-F: Functional Assessment of Chronic Illness Therapy Fatigue
FACT-G: Functional Assessment of Cancer Therapy General
EWB: Emotional Well-Being subscale
PCI: Perceived Cognitive Impairment
RNL: Reintegration to Normal Living Index
WCPA: Weekly Calendar Planning Activity
