# Supplementary material for: Characterizing changes in executive functions and performance in daily activities after chemotherapy: A pre-post mixed-methods study protocol
Source: PLoS One. 2024 Dec 13;19(12):e0314551. doi: 10.1371/journal.pone.0314551 (PMC11642909; doi:10.1371/journal.pone.0314551)
Supplement: S1 Protocol — (DOC) [file pone.0314551.s002.doc]

| **אפיון התפקודים הניהוליים והקשר לתפקוד היומיומי לפני ולאחר כימותרפיה בקרב אנשים עם סרטן**  **Characterizing the executive functioning and its association to daily functioning pre and following chemotherapy among individuals with cancer** |
| --- |

**Protocol No.:**

**Version: 1**

**Date of Protocol Version: 09.08.22**

**Sponsor:**

**Principal Investigator:** Dr. Abed Agbarya

**Secondary Investigator:**Dr. Khawla Loubani & Riham Shalbi, Occupational Therapists Occupational Therapy department Tel Aviv University.

| **אפיון התפקודים הניהוליים והקשר לתפקוד היומיומי לפני ולאחר כימותרפיה בקרב אנשים עם סרטן**  **Characterizing the executive functioning and its association to daily functioning pre and following chemotherapy among individuals with cancer** |
| --- |

**Approvals**

**Sponsor Signature: ________________________ Date: _______________**

**Principal Investigator Agreement:**

*I have carefully read and understood the provisions of this protocol and I am prepared to follow them in every detail in the conduct of this study.*

**Principal Investigator Signature: _____________ Date: ________________**

**Abstract**

**Background**: Cancer-related cognitive impairment (CRCI) is commonly experienced by individuals with non-central nervous system cancer due to the disease and treatment trajectory. CRCI has been mainly studied post chemotherapy and has found to include many cognitive impairments among them executive functions. Executive functions are high cognitive functions that include abilities such as (working memory, initiative, inhibition...). Impairments in executive functions can disrupt functioning and participation in complex daily activities such as cooking, driving that involve multitasking and coping with novel situations. These deficits impact person's persons social participation, work and quality of life, in addition to managing their medical condition after cancer. The association between objective and subjective cognitive complaints is still unclear and might be related to psychological aspects. In addition, there is currently no objective, functional, occupation-based assessment that detects the subtle mild cognitive changes particularly associated with CRCI. This raises the need for robust clinical testing of cognitive impairment in patients recently diagnosed with cancer, in order to guide clinicians in creating early individualized treatment plans. **Therefore, we aim to** explore changes in executive functioning and daily functioning following chemotherapy among individuals with non-central nervous system cancers. The specific aims are: 1) to compare executive functioning (objective and perceived), daily functioning, emotional status and fatigue pre versus post chemotherapy; 2) pre and post chemotherapy to determine which variables (executive functioning, emotional status, fatigue) best explain daily functioning. **Methods:** Fifty participants will be included aged between 18-75 years, newly diagnosed with a primary non-central nervous system malignancy (lung, breast, colon, ulcer, urinary tract, canine tumors) stages I-III pre- commencement of adjuvant (post-surgery) or neoadjuvant (prior to surgery) chemotherapy from the oncology department at Bnai-Zion Medical Center. A mixed-methods pre-post experimental design will be conducted. Pre and post chemotherapy cognitive and executive functions assessments and self-report measures will be performed by a qualified occupational therapist, will last up to one hour and will be performed in the oncology department of Bnai-Zion Medical Center. The primary outcome, namely "Functioning in daily activities" will be measured by the Canadian Occupational Performance Measure (COPM) an individualized semi-structured interview and outcome measure of performance and satisfaction with one's performance in daily activities. The secondary outcomes, namely Daily participation will be assessed by the Reintegration to Normal Living index (RNL) questionnaire; Working memory capacity will be assessed by the Backward Digit Span; Dual Tasking will be assessed by performing two tasks concurrently (motor by the Box & Blocks Test and cognitive by backward subtraction for 1 minute); Cognitive flexibility will be assessed by the Color Trail Test; Cognitive performance-based assessment will be assessed by the Weekly Calendar Planning Activity; Perceived cognitive executive functioning and Cognitive Self Efficacy will be assessed by the FACT-Cognitive Function in addition to three open questions; Emotional status will be assessed by the Emotional Well-Being FACT-G (General) questionnaire; Fatigue will be assessed by the Functional Assessment of Chronic Illness Therapy – Fatigue (FACIT-F); in addition to demographic and medical data questionnaire. **Procedure:** Following the approval of Bnai-Zion Medical Center, participants will be recruited by the help of their primary medical oncology staff. Each participant will undergo two assessment sessions; administered pre and post chemotherapy. Assessments will last up to one hour and will be performed in the oncology department of Bnai-Zion Medical Center. **Statistical analysis:** To compare between pre- and post-chemotherapy assessment: for normally distributed variables, paired t-tests or Wilcoxon Signed Ranks will be used; Multivariate linear regression analysis (Enter method) will be used to identify the variables that best explained daily performance (measured by COPM) pre and also post chemotherapy. *Qualitative data analysis* will include participant's answers to the open questions pre and post chemotherapy. Interviews will be analyzed using the phenomenological content analysis method.

**Introduction**

Following chemotherapy, persons living with and beyond cancer (LWBC) deal with short and long-term cognitive deficits (Dietrich and Kaiser, 2016). These deficits have been consistently reported across all cancer types, including cancers that are non-central nervous system (CNS) (Scherling and Smith, 2013) as cognitive related cancer impairment (CRCI). CRCI mainly studied after chemotherapy and includes impairments of short-term and working memory, attention, executive functions and/or processing speed (Hodgson et al., 2012; Li & Caeyenberghs, 2018; Lindner et al., 2014), deteriorated concentration, learning and language problems (Dietrich and Kaiser, 2016). Executive functions are higher cognitive abilities, which are crucial for performing complex daily activities (such as shopping, cooking or driving). Impairments in executive functions can disrupt complex activities and daily occupations that involve multitasking, strategic thinking, planning, and coping with novel situations (Katz & Maeir, 2011). These deficits impact person's occupational and social functioning and quality of life, maintaining work performance and carrying out daily tasks (Myers, 2013; Rust and Davis, 2013) in addition to managing their medical condition after cancer.

The impact of CRCI on everyday activities performance makes it an important area for occupational therapy, however it is relatively unexplored. This could be because neuropsychological tests do not adequately capture the deficits that participants experience in their everyday lives (Hodgson et al., 2013). CRCI is more commonly reported than objectively confirmed in persons living with or beyond cancer (LWBC) (Lange et al., 2019). Although the relation between objective (neuropsychological tests) and subjective (self-reports) complaints is still debated (Bender et al., 2006), self-report questionnaires might have more ecological validity compared to neuropsychological tests, occupational therapists can develop assessment tools and interventions to enable self-management and to enhance quality of life. However, there is currently no objective, functional, occupation-based assessment that detects the subtle mild cognitive changes particularly associated with CRCI.

Recent studies show that a significant portion of persons LWBC with non-central nervous system (CNS) tumors experience cognitive decline prior to treatment, suggesting a role for tumor-derived factors in modulating cognition and behavior (Olson & Marks, 2019). Cognitive complaints are often linked with psychological factors such as anxiety and depression (Menning et al., 2015). In addition to psychological factors also increased levels of fatigue accounted for 72% of the variance in perceived cognitive difficulties (Jenkins et al.,2016). Methodological issues may be a barrier in detecting cognitive deficits. For example, it has been noted that when using cross-sectional data, the inferred deficits may be inflated compared to longitudinal data because comparison to a pre-treatment baseline was not possible (Li & Caeyenberghs, 2018). Therefore, comparison to baseline assessment may allow to determine which impairments resulted from the treatment or was present prior to the commencement of treatment (Hodgson et al., 2013).

Recent clinical studies demonstrate that CRCI is an underappreciated symptom of cancer. These studies emphasize the need for robust clinical testing of cognitive impairment in persons recently diagnosed with cancer, in order to guide clinicians in creating early individualized treatment plans. Therefore, it is essential to characterize the cognitive functioning of adults newly diagnosed with cancer and its impact on patients' daily functioning in order to provide at an optimal timing intervention that aim to prevent cognitive deterioration and its effects on daily functioning.

**The overall aim of this study is** to explore changes in executive functioning and daily performance following chemotherapy among individuals with non-central nervous system cancers. The specific aims are: 1) to compare executive functioning (objective and perceived), daily performance, emotional symptoms and fatigue pre versus post chemotherapy; 2) pre and post chemotherapy to determine which variables (executive functioning, emotional, fatigue) best explain daily functioning

**Hypotheses according to aims:**

# **1a**. Post chemotherapy participant's objective and perceived executive functioning, emotional symptoms and fatigue will significantly worsen compared with pre chemotherapy.

# **2a**. Pre chemotherapy, daily functioning will be mostly explained by higher emotional symptoms and fatigue.

# **2b.** Post chemotherapy, daily functioning will be mostly explained by objective and perceived executive functioning deficits.

# **Participants**

Fifty participants will be included from the oncology department at Bnai-Zion Medical Center. *Inclusion criteria*: adults aged between 18-75 years, newly diagnosed with a primary non-central nervous system malignancy (lung, breast, colon, ulcer, urinary tract, canine tumors) stages I-III pre- commencement of adjuvant (post-surgery) or neoadjuvant (prior to surgery) chemotherapy, understand and read Hebrew*. Exclusion criteria*: participants diagnosed with central nervous system malignancies; had a previous malignancy, have: a metastatic disease, a history of any neurologic condition that could impair cognitive function (eg, dementia, stroke, brain injury), depression, or have 12 years of education (which could impair their performance on the neuropsychological evaluation). All eligible patients will provide written informed consent before enrollment.

**Study design**

A mixed-methods pre-post experimental design will be conducted.

The assessments will be performed by a qualified occupational therapist.

***Pre chemotherapy assessment***: will be conducted after diagnosis and before commencement of adjuvant or neoadjuvant chemotherapy.

***Post chemotherapy assessment***: will be performed after at least 3 months after commencement of adjuvant or neoadjuvant chemotherapy and within no more than one month after completing chemotherapy (before starting other treatments such as hormonal or biological therapies).

# **CRF and Measures (Appendix 1):**

1. The primary outcome ***"*** ***performance and satisfaction performance in daily activities "*** will be measured by the Canadian Occupational Performance Measure (COPM) (Law, et al., 1990); an individualized semi-structured interview and outcome measure of performance and satisfaction with one's performance in self-care, productive, leisure and social activities. During the pre-chemotherapy assessment participants identify up to five activities that they prioritize as meaningful for them. Each of these activities is then rated on a 10-point scale for perceived performance (1=not able to do at all, 10=able to do extremely well) and similarly for satisfaction with performance. The final scores of the COPM are the average scores for total performance and for satisfaction. During post chemotherapy assessment, the participants will repeat the COPM for the same activities they prioritized in the pre chemotherapy assessment. The COPM is valid and reliable (Carswell et al., 2004), widely used in research, and was used as the primary outcome measure in an RCT study that examined the effectiveness of an OT intervention with women after breast cancer (Loubani et al., 2022).
2. Secondary outcome measures:
3. *Daily participation* will be assessed by the Reintegration to Normal Living index (RNL; Wood-Dauphinée et al., 1988). The RNL is a questionnaire for evaluating the consequences of a disease and the required therapy on the patient’s life. The RNL has11 statements of participation in recreational and social activities, movement within the community, and degree of comfort the individual has in his/her role in the family and with other relationships. The responses are given on a 1–10-point Likert scale (1, highly do not agree; to 10 highly agree). The adjusted score is calculated as the (Total Score/110) × 100, and ranges from 10–100 points, with a higher score indicating more participation. The RNL has previously been used with patients undergoing surgery and treatment for rectal cancer (O’Connor et al., 2014).
4. Objective executive functioning:

- *Working memory* capacity will be assessed by the Backward Digit Span (**BDS**; Wechsler, 1997). The examiner mentions a series of digits in a forward order, thereafter the participant is asked to repeat the same series of digits in a backward order. The number of correct sequences will be recorded.

- *Dual Tasking* will be assessed by performing two tasks concurrently (motor and cognitive). For the motor task, we will use the Box & Blocks Test (BBT; Mathiowetz et al., 1985), a standardized assessment that has been widely used to assess manual dexterity, which is an important component of functional capacity (Canny et al., 2009; Hebert et al., 2012). The participant is asked to grasp and move 1-inch square wooden blocks from one side of an 8-in square box over a wooden partition to the other side within one-minute. The number of blocks transferred in 1 minute is registered. This test will be performed first by the dominant and then by the non-dominant hands. Lower scores indicate greater upper-extremity impairments. The BBT's psychometric properties have been well established in several adult populations (Kontson et al., 2017). For the single cognitive task, the participants will be asked to count by subtracting a number (e.g., 7 beginning from 399) and then to keep subtracting the same number from his/her answer in 1 minute. The order of the dual task assessment will be the following order (1 minute for each task): single cognitive task, single motor task with dominant hand, single motor task with non-dominant hand, dual motor-cognitive task with dominant hand, dual motor-cognitive task with non-dominant hand.

- *Cognitive flexibility* will be assessed by the Color Trail Test (**CTT**; D’Elia et al., 1996) a paper and pencil test of visual attention timed neuropsychological test. The CTT 1 consists of 25 circled numbers from 1 to 25 (even numbers in a yellow background and odd numbers in a pink background). The participant will be asked to rapidly connect the circles in consecutive order. The CTT2 consists of double the stimuli as the CTT1 with two sets of the 25 numbers in each color (pink and yellow). The participant will be asked to connect the numbers in ascending order alternating between the two-color sets. Scoring is calculated by measuring completion time (up to 240 seconds).

c. *Cognitive performance-based* assessment will be assessed by the Weekly Calendar Planning Activity (**WCPA**; Toglia, 2015). WPCA is a paper-and-pencil, performance-based

assessment that examines the influence of subtle executive function difficulties on person’s ability to perform a multiple-step activity. A randomly ordered list of 17 appointments is presented to the participant who is asked to schedule them using a 1-week calendar.

while adhering to five rules Scores include measures of accuracy, number of appointments entered, planning time (time from the beginning of the assessment until the first appointment is scheduled), total time, efficiency (calculated from the accuracy and total time), error types, number of rules followed out of the five given, and number of strategies used.

d. Perceived cognitive executive functioning

- *Cognitive Self Efficacy* (**SE**) will be assessed by the Cognitive Self Abilities Scale CogPCA (FACT-Cognitive Function Version 3). [The](https://www.facit.org/) CogPCA scale includes 7 statements with responses given on a 5-point Likert scale (from 0, never; to 4, several times a day). The score range= 0–28 with higher scores indicating higher difficulties.

*- Perceived Cognitive Impairment* will be assessed by the Perceived Cognitive Impairment scale – PCI (FACT-Cognitive Function Version 3; Wagner et al., 2009). The PCI scale includes 18 statements with responses given on a 5-point Likert scale (from 0, never; to 4, several times a day). The score range= 0–72 with higher scores indicating higher difficulties.

*- Three open-ended questions* (pre chemo "since diagnosis" and post chemo "since treatment"). The questions regarding person's changes in cognitive functioning and the consequences of these changes on his/her daily functioning will be presented on a sheet of paper and they will be asked to write their answers bellow together with the accessor.

1. *Emotional status* will be assessed by the Emotional Well-Being subscale (EWB; Cella et al., 1993) from the FACT-G (General) questionnaire (https://www.facit.org/). The EWB scale includes six statements with responses given on a 5-point Likert scale (from 0, not at all; to 4, very much). The score range= 0–24 with higher scores indicating higher emotional deficits.
2. *Fatigue* will be assessed by the Functional Assessment of Chronic Illness Therapy – Fatigue (FACIT-F) (Version 4; <https://www.facit.org/> ). The FACT Fatigue includes 13 statements with responses given on a 5-point Likert scale (from 0, not at all; to 4, very much). The score range= 0-52 with higher scores indicating more fatigue.
3. *Demographic and medical data Questionnaire* will include the following details: age, education, sex, marital status, number of children, years of education, work status, dominant hand, socioeconomic level (income level), type/stage of cancer, disease characteristics (e.g., size of tumor, number of affected lymph nodes), details of treatments received (e.g., types of surgery chemotherapy regimens, date of chemo commencement, number of cycles completed.

In addition, participants will be asked if we can contact them in the near future to perform a semi-structured interview: "do you agree to participate in an additional individual interview in the future as part of this study?"

**Procedure**

Following the approval of Bnai-Zion Medical Center, participants will be recruited by the help of their primary medical oncology staff. Each participant will undergo two assessment sessions; administered pre and post chemotherapy. Assessments will last up to one hour and will be performed in the oncology department of Bnai-Zion Medical Center or at the participant's home, at their choice. In addition, in cases where the assessment was not completed due to time constraints, the questionnaires will be completed remotely with the participant via ZOOM. All assessments will be conducted by a qualified occupational clinic.

#

# **Statistical analysis**

# *Quantitative data analysis* will be performed by SPSS version 27. Descriptive statistics will be used to describe the study population and outcome measures. Normal distribution of the variables will be verified using Shapiro Wilk test. To compare between pre- and post-chemotherapy assessment: for normally distributed variables, paired t-tests will be used; when data are not distributed normally and scales of measurements are ordinal, non-parametric tests will be used; for the primary outcome (COPM, an ordinal scale), the Wilcoxon Signed Ranks test will be used.

# Multivariate linear regression analysis (Enter method) will be used to identify the variables that best explained daily performance (measured by COPM) pre and also post chemotherapy. Variables (e.g., cognitive self-efficacy, fatigue, emotional status, executive functioning) that will be significantly correlated to daily performance (pre / post) will be entered into the model. Scatter plots of residuals against the model data will be inspected, as well as outliers and influential data points, and the variance inflation factor for multicollinearity. All analyses will be conducted using SPSS for Windows version 25.0 (SPSS, Inc., Chicago, IL, USA).

# *Qualitative data analysis* will include participant's answers to the open questions pre and post chemotherapy. Interviews will be analyzed using the phenomenological content analysis method. Data will be coded and categorized into groups to identify major themes. Two authors will perform the text analysis inductively and undertake thick description and validation by a few participants to ensure trustworthiness (Creswell & Poth, 2016).

**References**

Bender C. M., Sereika S. M., Berga S. L., Vogel, V.G., Brufsky A.M., Paraska K.K. (2006). Cognitive impairment associated with adjuvant therapy in breast cancer. *Psychooncology 15*, 422–30

Canny M. L., Thompson J. M., Wheeler, M. J. (2009). Reliability of the box and block test of manual dexterity for use with patients with fibromyalgia. *American Journal of Occupational Therapy; 63*(4):506–10. PMID: 19708480

Carswell, A., McColl, M., Baptiste, S., Law, M., Polatajko, H., & Pollock, N. (2004). The Canadian Occupational Performance Measure: A research and clinical literature review. *Canadian Journal of Occupational Therapy, 71* (4), 210-222.

Creswell, J. W., & Poth, C. N. (2016). Qualitative inquiry and research design: Choosing among five approaches. Sage

D’Elia L.F., Satz P, Uchiyama CL, White T. Color Trails Test; Odessa. Psychological Assessment Resources, FL 1996

Dietrich, J., Prust, M., & Kaiser, J. (2015). Chemotherapy, cognitive impairment and hippocampal toxicity. Neuroscience, 309, 224-232.‏

Wagner, L., Sweet, J., Butt, Z., Lai, J.-S., & Cella, D. Measuring patient self-reported cognitive function: development of the functional assessment of cancer therapy–cognitive function instrument. Journal of Supportive Oncology 2009; 7(6): W32-W39.

Cella D.F., Tulsky D.S., Gray G., Sarafian B., Lloyd S., Linn E., Bonomi A., Silberman M., Yellen S.B., Winicour P., Brannon J., Eckberg K., Purl S., Blendowski C., Goodman M., BarnicleM., Stewart I., McHale M., Bonomi P., Kaplan E., Taylor S., Thomas C., Harris J. The Functional Assessment of Cancer Therapy (FACT) Scale: Development and validation of the general measure. *Journal of Clinical Oncology*1993; 11(3): 570-579.

Functional Assessment of Chronic Illness Therapy – Fatigue (FACIT-F) (Version 4). Retrieved 27.07.22 from https://www.facit.org/.

Hebert JS, Lewicke J. Case report of modified Box and Blocks test with motion capture to measure prosthetic function. Journal of Rehabilitation Research & Development. 2012; 49(8):1163–74.

Hodgson, K. D., Hutchinson, A. D., Wilson, C. J., & Nettelbeck, T. (2013). A meta-analysis of the effects of chemotherapy on cognition in patients with cancer. Cancer treatment reviews, 39(3), 297-304.‏

Jenkins, V., Thwaites, R., Cercignani, M., Sacre, S., Harrison, N., Whiteley-Jones, H., et al., 2016. A feasibility study exploring the role of pre-operative assessment when examining the mechanism of’ chemo-brain’ in breast-cancer patients. Springerplus 5, 390. <http://dx.doi.org/10.1186/s40064-016-2030-y>

Katz, N., & Maeir, A. (2011). Higher-level cognitive functions enabling participation: Awareness and executive functions. In N. Katz (Ed.), Cognition, occupation, and participation across the life span: Neuroscience, neurorehabilitation, and models of intervention in occupational therapy (pp. 13–40). Bethesda, MD: AOTA Press.

Kontson K, Marcus I, Myklebust B, Civillico E (2017) Targeted box and blocks test: Normative data and comparison to standard tests. PLoS ONE 12(5): e0177965. <https://doi.org/10.1371/journal.pone.0177965>

Lange M, Licaj I, Clarisse B et al. (2019). Cognitive complaints in cancer survivors and expectations for support: results from a web-based survey. Cancer Med; 8(5): 2654–2663.

Law, M., Baptiste, S., McColl, M., Opzoomer, A., Polatajko, H., & Pollock, N. (1990). The Canadian occupational performance measure: an outcome measure for occupational therapy. Canadian Journal of Occupational Therapy, 57(2), 82-87.‏

Li, M., & Caeyenberghs, K. (2018). Longitudinal assessment of chemotherapy-induced changes in brain and cognitive functioning: A systematic review. *Neuroscience & Biobehavioral Reviews*, *92*, 304-317.‏

Lindner O., C, Phillips B, McCabe M., G., et al. (2014). A meta-analysis of cognitive impairment following adult cancer chemotherapy. Neuropsychology; 28(5): 726–740

Mathiowetz V, Volland G, Kashman N, Weber K (1985). Adult norms for the Box and Block Test of manual dexterity. American Journal of Occupational Therapy, 39:386–391. doi: 10.5014/ajot.39.6.386

Menning, S., de Ruiter, M.B., Veltman, D.J., Koppelmans, V., Kirschbaum, C., Boogerd, W., et al., 2015. Multimodal MRI and cognitive function in patients with breast cancer prior to adjuvant treatment—the role of fatigue. Neuroimage: Clin. 7, 547–554

Myers, J. S. (2013). Cancer- and chemotherapy-related cognitive changes: The patient experience. Seminars in Oncology Nursing, 29, 300–307

O’Connor, G., Coates, V., & O’Neill, S. (2014). Randomised controlled trial of a tailored information pack for patients undergoing surgery and treatment for rectal cancer. European Journal of Oncology Nursing, 18(2), 183-191.‏

Olson, B., & Marks, D. L. (2019). Pretreatment cancer-related cognitive impairment—Mechanisms and outlook. Cancers, 11(5), 687.‏

DPh, M. S. W. (2013). Chemobrain in underserved African American breast cancer survivors: A qualitative study. Clinical Journal of Oncology Nursing, 17(2), E29.‏

Scherling, C. S., & Smith, A. (2013). Opening up the window into “chemobrain”: a neuroimaging review. Sensors, 13(3), 3169-3203.‏

Toglia, J. (2015). Weekly calendar planning activity: A performance test of executive function. Bethesda, MD: AOTA Press. Retrieved from https://myaota.aota.org/shop_aota/prodview. aspx?TYPE=D&PID=271083346&SKU=900369

Wechsler, D. (1997). Wechsler Adult Intelligence Scale (3rd ed.). San Antonio, TX: Psychological Corporation.

Wood-Dauphinée SL, Opzoomer MA, Williams JI, Marchand B, Spitzer WO. Assessment of global function: the Reintegration to Normal Living Index. Arch Phys Med Rehabil 1988; 69:583-90


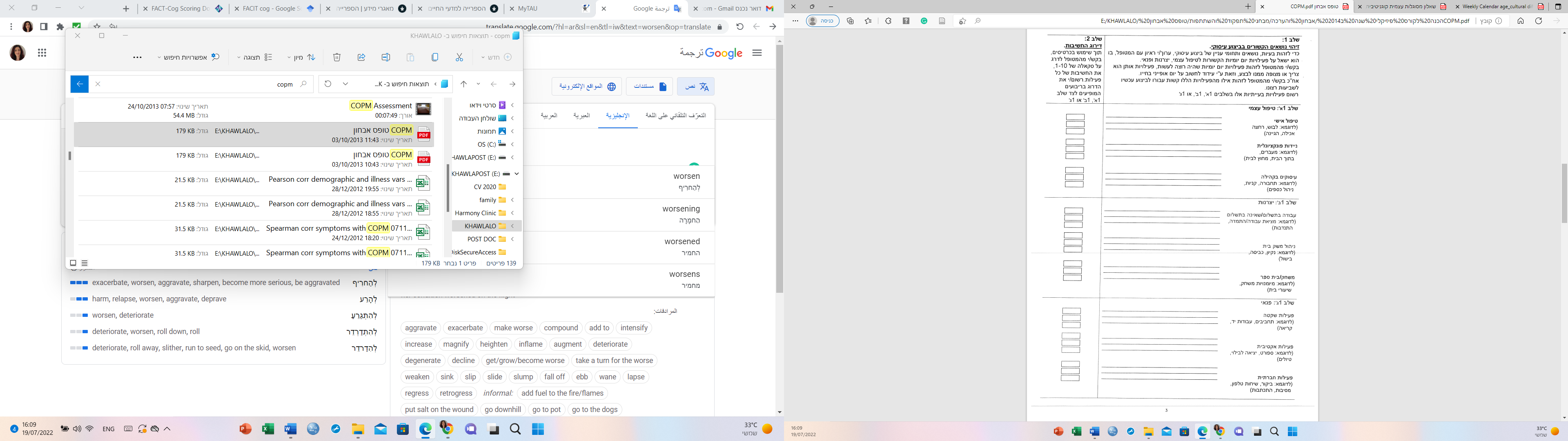


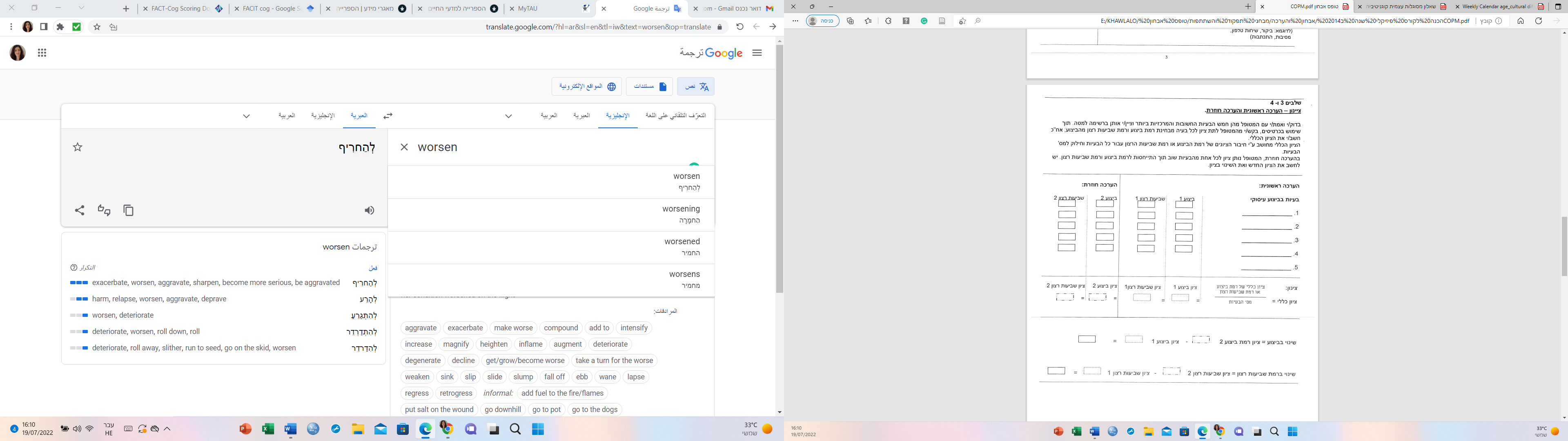


**Backward Digit Span (BDS; Wechsler, 1997)**

**נבדק מספר_____**

במטלה הבא את/ה מתבקש/ת לחזור בסדר הפוך על רצף הספרות הבאות:

עוצרים את המטלה כאשר יש שני X ברצף.

|  | נסיון ראשון | √ או X | נסיון שני | √ או X | סה"כ |
| --- | --- | --- | --- | --- | --- |
| א. | 83 |  | 29 |  |  |
| ב. | 475 |  | 615 |  |  |
| ג. | 2619 |  | 3852 |  |  |
| ד. | 28736 |  | 59413 |  |  |
| ה. | 624719 |  | 276391 |  |  |
| ו. | 4183627 |  | 1586937 |  |  |
| ז. | 52624197 |  | 94617385 |  |  |
|  |  |  |  | סה"כ למבחן: | |

Box & Blocks Test (BBT; Mathiowetz et al.,1985)
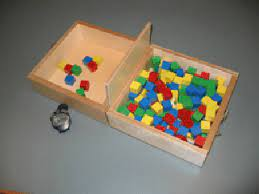


Color Trail Test (**CTT**; D’Elia et al., 1996)


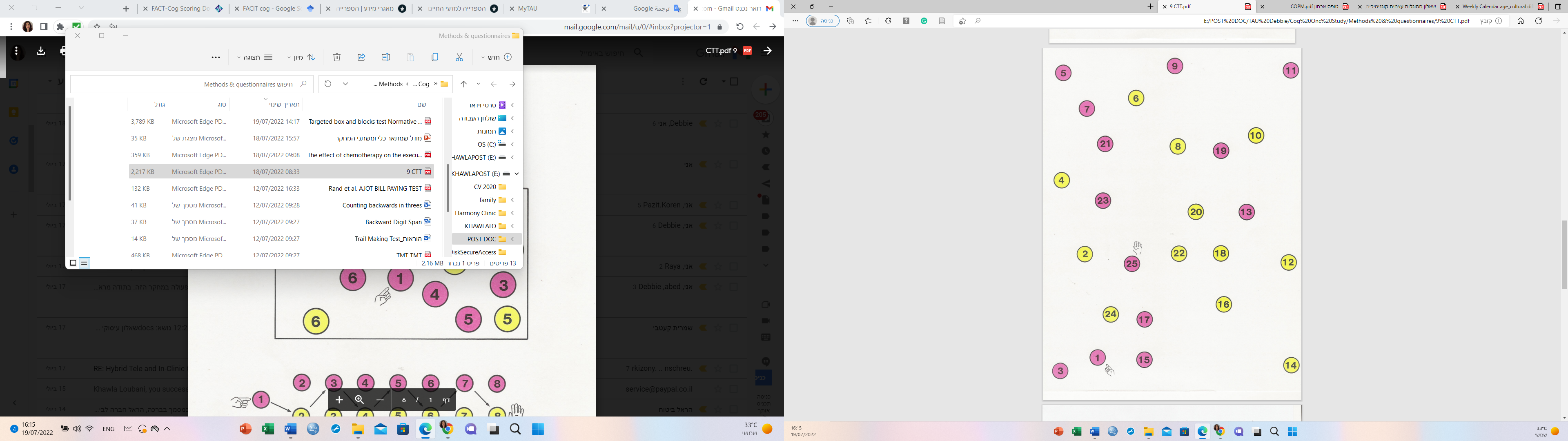


Weekly Calendar Planning Activity (**WCPA**; Toglia, 2015)


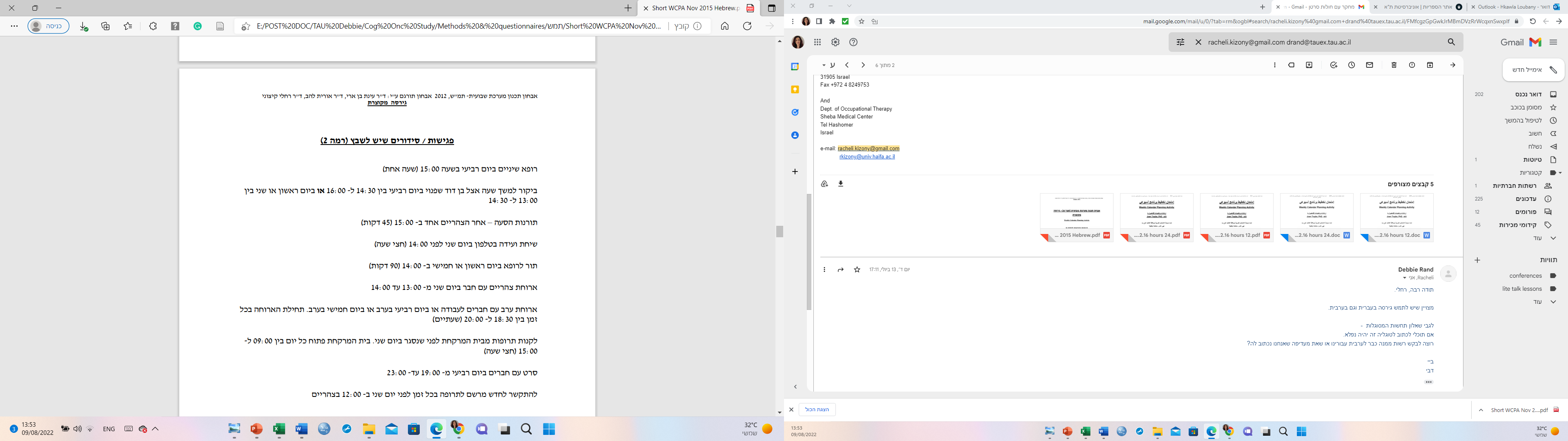


תפקוד קוגניטיבי (גרסה 3)- FACT

לפניך רשימה של משפטים שנקבעו כחשובים על ידי אנשים שנמצאים במצב דומה לשלך. **הקף/הקיפי בעיגול, או סמן/סמני באופן אחר מספר אחד בלבד בכל שורה כדי לציין את תשובתך, תוך התייחסות ל-7 הימים האחרונים.**

|  | **תפיסת הליקויים הקוגניטיביים** | אף פעם | בערך פעם בשבוע | פעמיים עד שלוש בשבוע | כמעט כל יום | מספר פעמים ביום |
| --- | --- | --- | --- | --- | --- | --- |
| CogA1 | **יש לי קושי לגבש מחשבות** 0 | 0 | 1 | 2 | 3 | 4 |
| CogA3 | החשיבה שלי איטית 0 | 0 | 1 | 2 | 3 | 4 |
| CogC7 | יש לי קושי להתרכז 0 | 0 | 1 | 2 | 3 | 4 |
| CogM9 | יש לי קושי למצוא את הדרך למקומות מוכרים 0 | 0 | 1 | 2 | 3 | 4 |
| CogM10 | **יש לי קושי לזכור היכן הנחתי את החפצים שלי, כמו המפתחות שלי או הארנק שלי** 0 | 0 | 1 | 2 | 3 | 4 |
| CogM12 | יש לי קושי לזכור מידע חדש, כמו מספרי טלפון או הוראות פשוטות 0 | 0 | 1 | 2 | 3 | 4 |
| CogV13 | **יש לי קושי להיזכר בשם של חפץ בזמן שאני מדבר/ת עם מישהו** | 0 | 1 | 2 | 3 | 4 |
| CogV15 | **יש לי קושי למצוא את המילה הנכונה כדי להביע את עצמי** 0 | 0 | 1 | 2 | 3 | 4 |
| CogV16 | אני משתמש במילה הלא נכונה כאשר אני מתכוון לחפץ מסוים 0 | 0 | 1 | 2 | 3 | 4 |
| CogV17b | יש לי קושי לומר את מה שאני מתכוון/ת בשיחה עם אחרים 0 | 0 | 1 | 2 | 3 | 4 |
| CogF19 | אני נכנס/ת לחדר ושוכח/ת מה התכוונתי להביא או לעשות שם 0 | 0 | 1 | 2 | 3 | 4 |
| CogF23 | **אני צריך/ה להתאמץ כדי להתרכז במה שאני עושה אחרת אני עושה טעות** 0 | 0 | 1 | 2 | 3 | 4 |
| CogF24 | אני שוכח/ת את שמותיהם של אנשים מעט אחרי שהציגו לי אותם 0 | 0 | 1 | 2 | 3 | 4 |

**הקף/הקיפי בעיגול, או סמן/סמני באופן אחר מספר אחד בלבד בכל שורה כדי לציין את תשובתך, תוך התייחסות ל-7 הימים האחרונים.**

|  |  | אף פעם | בערך פעם בשבוע | פעמיים עד שלוש בשבוע | כמעט כל יום | מספר פעמים ביום |
| --- | --- | --- | --- | --- | --- | --- |
| CogF25 | התגובות שלי במצבים יומיומיים איטיות 0 | 0 | 1 | 2 | 3 | 4 |
| CogC31 | עלי להתאמץ יותר מהרגיל כדי לעקוב אחרי מה שאני עושה 0 | 0 | 1 | 2 | 3 | 4 |
| CogC32 | החשיבה שלי איטית יותר מהרגיל | 0 | 1 | 2 | 3 | 4 |
| CogC33a | עלי להתאמץ יותר מהרגיל כדי להביע את עצמי באופן ברור 0 | 0 | 1 | 2 | 3 | 4 |
| CogC33c | עלי להשתמש ברשימות כתובות לעיתים קרובות יותר מהרגיל כדי שלא אשכח דברים 0 | 0 | 1 | 2 | 3 | 4 |
| CogMT1 | **יש לי קושי לעקוב אחרי מה שאני עושה אם קוטעים אותי** 0 | 0 | 1 | 2 | 3 | 4 |
| CogMT2 | **יש לי קושי** לעבור הלוך ושוב בין משימות שונות הדורשות חשיבה 0 | 0 | 1 | 2 | 3 | 4 |

**הקף/הקיפי בעיגול, או סמן/סמני באופן אחר מספר אחד בלבד בכל שורה כדי לציין את תשובתך, תוך התייחסות ל-7 הימים האחרונים.**

|  | תפיסת היכולות הקוגניטיביות | כלל לא | **מעט** | במידה מסוימת | במידה רבה | במידה רבה  מאוד |
| --- | --- | --- | --- | --- | --- | --- |
|  |
| CogPC1 | אני מסוגל/ת להתרכז | 0 | 1 | 2 | 3 | 4 |
| CogPV1 | אני מסוגל/ת לחשוב על מילים שאני רוצה להשתמש בהן כאשר אני מדבר/ת עם מישהו | 0 | 1 | 2 | 3 | 4 |
| CogPM1 | אני מסוגל/ת לזכור דברים, כמו איפה הנחתי את המפתחות או הארנק שלי | 0 | 1 | 2 | 3 | 4 |
| CogPM2 | אני מסוגל/ת לזכור לבצע דברים, כמו לנטול תרופה או לקנות משהו שאני צריך/ה | 0 | 1 | 2 | 3 | 4 |
| CogPF1 | אני מסוגל/ת לשים לב ולעקוב אחרי מה שאני עושה בלי מאמץ נוסף | 0 | 1 | 2 | 3 | 4 |
| CogPCH1 | המח שלי חד כפי שהיה תמיד | 0 | 1 | 2 | 3 | 4 |
| CogPCH2 | הזיכרון שלי טוב כפי שהיה תמיד | 0 | 1 | 2 | 3 | 4 |
| CogPMT1 | אני מסוגל/ת לעבור הלוך וחזור בין שתי משימות הדורשות חשיבה | 0 | 1 | 2 | 3 | 4 |
| CogPMT2 | אני מסוגל/ת לעקוב אחרי מה שאני עושה גם אם מפריעים לי | 0 | 1 | 2 | 3 | 4 |

Reintegration to Normal Living index (RNL; Wood-Dauphinée et al., 1988)


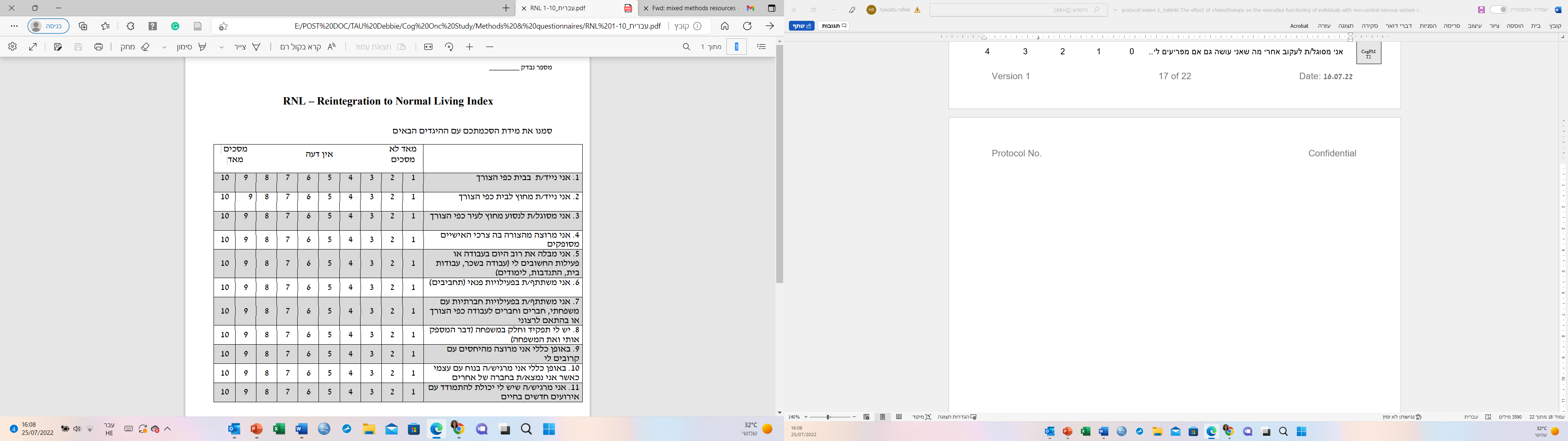


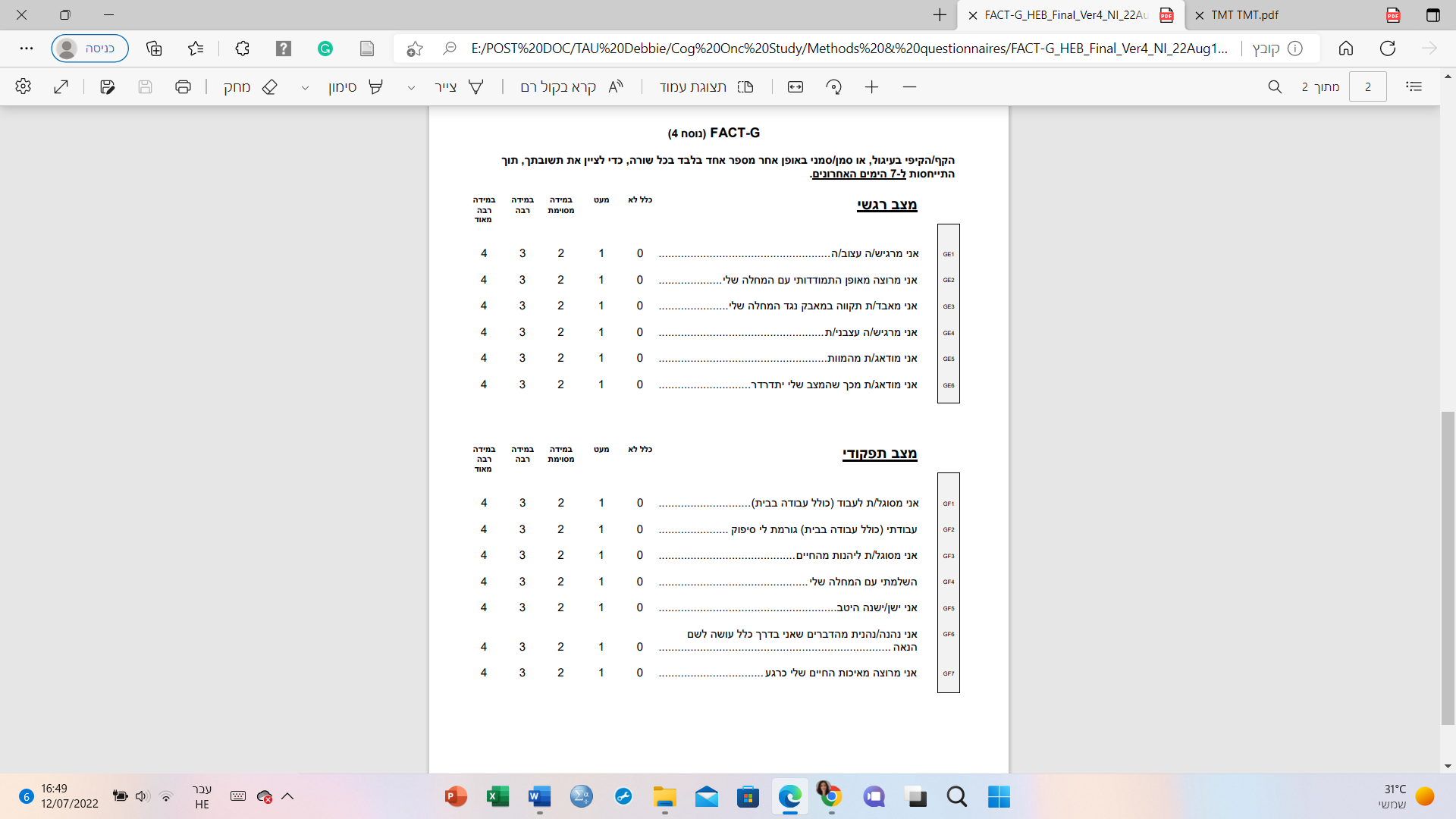


**FACT Fatige**

לפניך רשימה של משפטים שנקבעו כחשובים על ידי אנשים שלוקים באותה מחלה שיש לך. **הקף/הקיפי בעיגול, או סמן/סמני באופן אחר מספר אחד בלבד בכל שורה כדי לציין את תשובתך, תוך התייחסות ל-7 הימים האחרונים.**

| במידה רבה  מאוד | במידה  רבה | במידה  מסוימת | מעט | כלל לא |  |  |
| --- | --- | --- | --- | --- | --- | --- |
| 4 | 3 | 2 | 1 | 0 | אני מרגיש/ה תשוש/ה | HI7 |
| 4 | 3 | 2 | 1 | 0 | אני מרגיש/ה חולשה בכל הגוף | HI12 |
| 4 | 3 | 2 | 1 | 0 | אני מרגיש/ה אדישות לכל דבר | An1 |
| 4 | 3 | 2 | 1 | 0 | אני חש/ה עייפות | An2 |
| 4 | 3 | 2 | 1 | 0 | קשה לי להתחיל דברים בגלל שאני עייף/ה | An3 |
| 4 | 3 | 2 | 1 | 0 | קשה לי לסיים דברים בגלל שאני עייף/ה | An4 |
| 4 | 3 | 2 | 1 | 0 | יש לי אנרגיה | An5 |
| 4 | 3 | 2 | 1 | 0 | אני מסוגל/ת לבצע את פעילויותיי הרגילות | An7 |
| 4 | 3 | 2 | 1 | 0 | אני זקוק/ה לשינה במשך היום | An8 |
| 4 | 3 | 2 | 1 | 0 | אני עייף/ה מדי כדי לאכול | An12 |
| 4 | 3 | 2 | 1 | 0 | אני צריך/ה עזרה בביצוע פעילויותיי הרגילות | An14 |
| 4 | 3 | 2 | 1 | 0 | אני מתוסכל/ת מהיותי עייף/ה מדי כדי  לבצע את הדברים שאני רוצה לעשות | An15 |
| 4 | 3 | 2 | 1 | 0 | אני נאלץ/ת להגביל את הפעילות החברתית  שלי בגלל שאני עייף/ה | An16 |

**שאלות פתוחות: הערכה מס' (1) לפני כימותרפיה – דף למילוי יחד עם הנבדק/ת**

1. תכף אעביר לך כמה אבחונים שבודקים קוגניציה.

לפני שנתחיל, האם תוכל/י לתאר איך הולך לך עם מטלות קוגניטיביות מורכבות? *האם יש משהו שאת/ה יותר או פחות אוהב/ת?*

_______________________________________________________________________________________________________________________________________________________________________________________________________________________________________________________________

_____________________________________________________________________________________ **ב. אם התשובה מעידה על קושי**

ב1. נא פרט/י מהם הקשיים? מה האתגרים? למה לא הולך? נא לפרט.

______________________________________________________________________________________________________________________________________________________________________________

_______________________________________________________________________________________

_______________________________________________________________________________________

ב2. ממה נובע/ים קשיים אלה לדעתך? נא לפרט.

____________________________________________________________________________________________________________________________________________________________________________________________________________________________________________________________________________________________________________________________________________________________

ב3. כיצד לדעתך קשיים אלה באים לידי ביטוי בתפקודך היומי? נא לפרט.

____________________________________________________________________________________________________________________________________________________________________________________________________________________________________________________________________________________________________________________________________________________

ב3. איזה עוד גורמים לדעתך משפיעים על התפקוד היומיומי שלך? נא לפרט.

____________________________________________________________________________________________________________________________________________________________________________________________________________________________________________________________________________________________________________________________________________________

**ג. אם התשובה אינה מעידה על קשיים**

ג1. איזה עוד גורמים לדעתך משפיעים על התפקוד היומיומי שלך? נא לפרט.

____________________________________________________________________________________________________________________________________________________________________________________________________________________________________________________________________________________________________________________________________________________

**שאלות פתוחות: הערכה מס' (2) אחרי כימותרפיה – דף למילוי יחד עם הנבדק/ת**

1. בדומה לפגישה הקודמת, תכף אעביר לך כמה אבחונים שבודקים קוגניציה.

לפני שנתחיל, האם תוכל/י לתאר איך הולך לך עם מטלות קוגניטיביות מורכבות? *האם יש משהו שאת/ה יותר או פחות אוהב/ת?*

_______________________________________________________________________________________________________________________________________________________________________________________________________________________________________________________________

_____________________________________________________________________________________ **ב. אם התשובה מעידה על קושי**

ב1. מהם השינויים שאת/ה חווה במצבך הקוגניטיבי? נא לפרט.

______________________________________________________________________________________________________________________________________________________________________________

_______________________________________________________________________________________

_______________________________________________________________________________________

ב2. ממה נובע/ים שינויים אלה לדעתך? נא לפרט.

____________________________________________________________________________________________________________________________________________________________________________________________________________________________________________________________________________________________________________________________________________________________

***אם לא מעלה כימותרפיה כגורם, לשאול: *יש אנשים שמדווחים על שינוי בקוגניציה בעקבות כימותרפיה. האם אתה מרגיש שינויים כאלה? אם כן נא לפרט***

*____________________________________________________________________________________________________________________________________________________________________________________________________________________________________________________________________________________________________________________________________________________________*

ב3. כיצד לדעתך שינויים אלה באים לידי ביטוי בתפקודך היומי? נא לפרט.

____________________________________________________________________________________________________________________________________________________________________________________________________________________________________________________________________________________________________________________________________________________

ב3. איזה עוד גורמים לדעתך משפיעים על התפקוד היומיומי שלך? נא לפרט.

____________________________________________________________________________________________________________________________________________________________________________________________________________________________________________________________________________________________________________________________________________________

***אם לא מעלה כימותרפיה כגורם, לשאול: יש אנשים שמדווחים על שינוי בתפקוד היומיומי בעקבות כימותרפיה. האם אתה מרגיש שינויים כאלה? אם כן נא לפרט**

**___________________________________________________________________________________________________________________________________________________________________________________________________________________________________________________________________________________________________________________________________________________________________________________________________________**

**ג. אם התשובה אינה מעידה על קשיים**

ג1. איזה עוד גורמים לדעתך משפיעים על התפקוד היומיומי שלך? והאם תוכל/י לפרט?

____________________________________________________________________________________________________________________________________________________________________________________________________________________________________________________________________________________________________________________________________________________

**שאלות פתוחות: הערכה מס' (1) לפני כימותרפיה – דף עזר למעריכה**


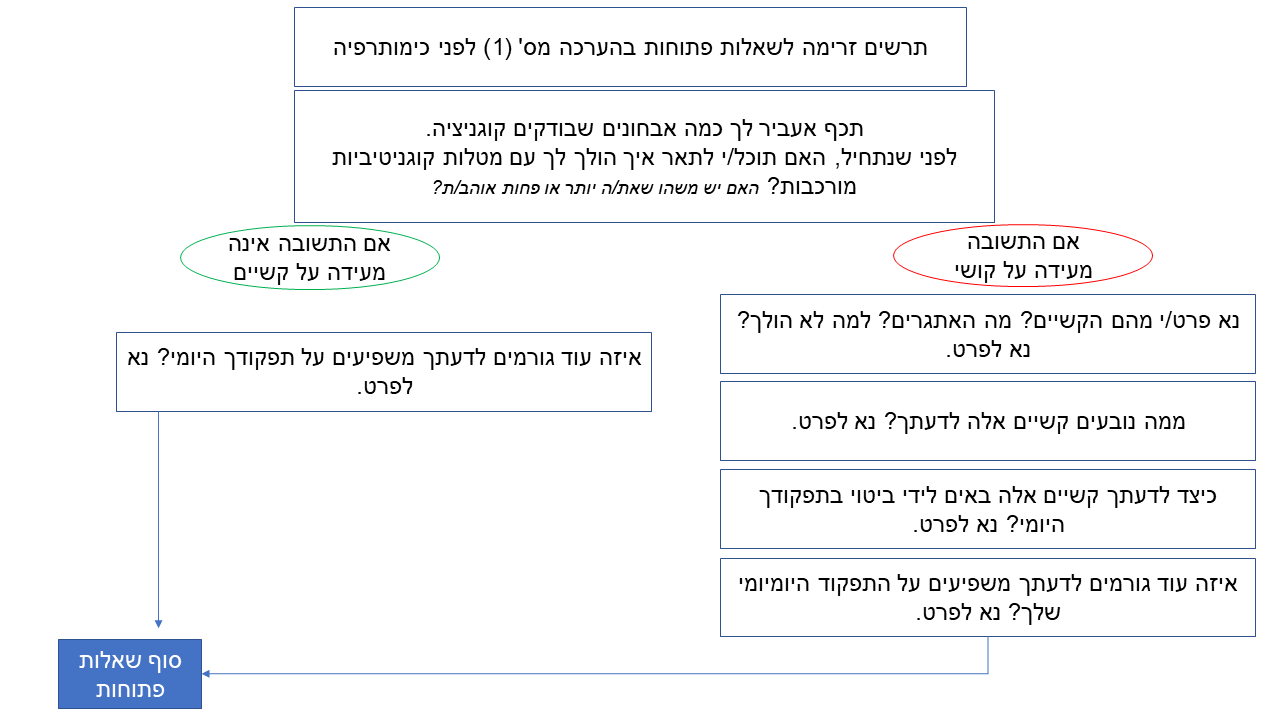


**שאלות פתוחות: הערכה מס' (2) אחרי כימותרפיה – דף עזר למעריכה**


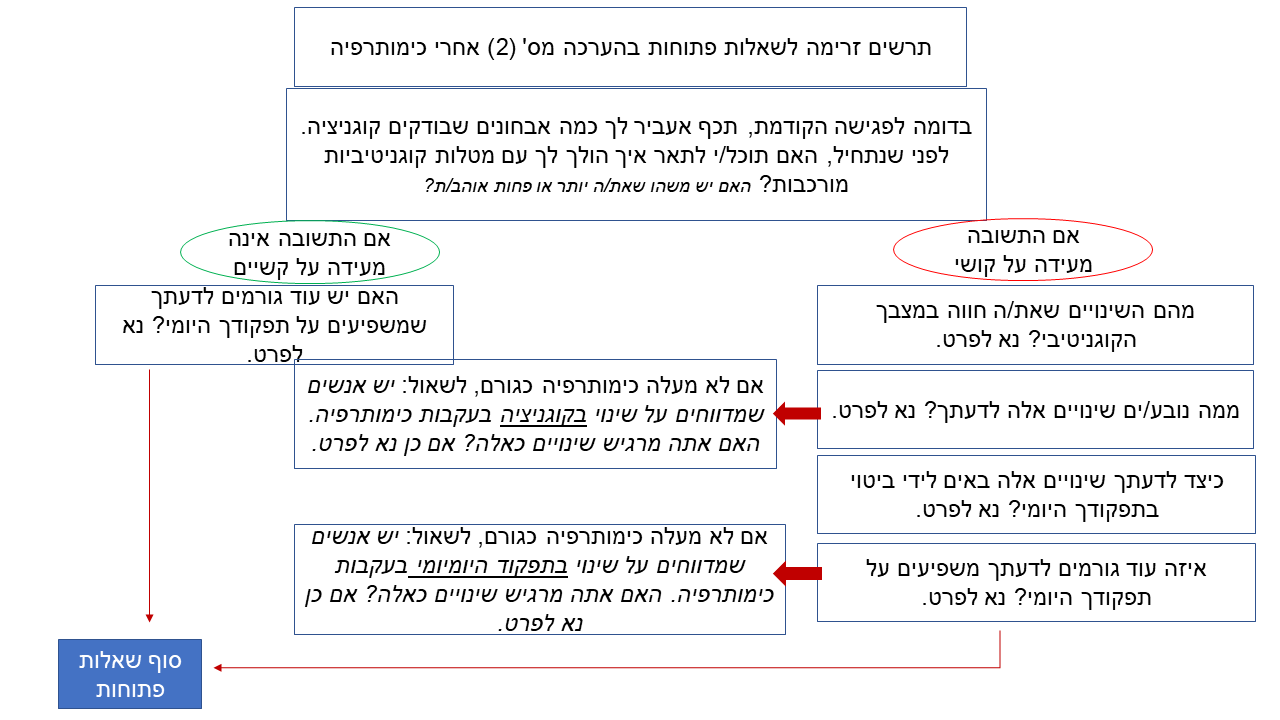


**שאלון דמוגרפי ונתוני מחלה**

**א. נתונים אישיים של הנבדקת Demographic data**

1. גיל Age (שנים ) __ __

2. מצב משפחתי Marital status:

א. רווק/ה ב. נשואי/ה (ללא ילדים) ג. נשואי/ה + _____ ד. גרוש/ה ה. אלמנ/ה.

3. מספר שנות לימודים Yrs of study: ________

4. מה הוא המקצוע שלך Profession ? ___________________

5. מצב תעסוקתי Work status : א. שכירה ב. עצמאית ג. סטודנטית

6. היקף משרה לפני המחלה Job scope pre : א. משרה מלאה ב. משרה חלקית

7. האם הורדת באחוז המשרה בעקבות המחלה : א. כן ב. לא

8. הכנסה חודשית שלך מהעבודה Month income:

א. נמוכה מאוד ב. נמוכה ג. בינונית ד.גבוהה ה. גבוהה מאוד.

עד 2000 ₪ עד 3000 ₪ עד 6000 ₪ מעל 6000 ₪ מעל 11000 ₪

9. יד דומיננטית: א. ימין ב. שמאל

**פרטים רפואיים – מחלה וסימפטומים**

1. אבחנה Diagnoses: _________________________________________________

2. דרגת סרטן: __________________________________________

3. תאריך אבחנה? ____________.

4. האם עברת ניתוח? א. כן, פרט/י________________ ב. לא

5. מה סוג/י הטיפול הכימותרפי : __________________________________

7. תאריך תחילת טיפול כימותרפי:____________________

8. כמה טיפולים מתוכננים?____________________

9. האם את מקבלת טיפולים נוספים (עוס"ית/ פיזיותרפיה, וכו'...) Therapies other?

א. כן, פרט/י_________________ ב. לא

10. האם אתה מסכים/ה להשתתף בראיון אישי בעתיד במסגרת מחקר זה?

א. כן ב. לא
